# Lateralization of Color Discrimination Performance and Lexical Effects in Patients With Chronic Schizophrenia

**DOI:** 10.3389/fnhum.2021.702086

**Published:** 2021-09-28

**Authors:** Tomohiro Kogata, Tetsuya Iidaka

**Affiliations:** ^1^Department of Physical and Occupational Therapy, Graduate School of Medicine, Nagoya University, Nagoya, Japan; ^2^Brain and Mind Research Center, Nagoya University, Nagoya, Japan

**Keywords:** color perception, categorical perception, language, laterality, visual field, reaction time

## Abstract

**Introduction:** Patients with schizophrenia experience various visual disturbances. However, information regarding color perception in these patients is rare. In this study, we used a lateralized color search task to investigate whether difference in color name affects color recognition in patients with schizophrenia.

**Methods:** In a color search task, we controlled the position of the target that emerged from the left visual field (LVF) or right visual field (RVF) as well as the color category. In this task, both the target and the distractors had the same or different color name (e.g., blue or green).

**Results:** Patients with schizophrenia showed faster performance in the color search task with different color names for target-distractors when the target emerged from the LVF than when it emerged from the RVF. However, the same laterality was not observed in healthy controls. This finding indicates that semantic processing for color name differences influenced visual discrimination performance in patients with schizophrenia more profoundly in the LVF than in the RVF.

**Conclusion:** This lateralized performance could imply the failure of the left hemisphere language processing dominance in schizophrenia. A search paradigm combining target position and category may indicate that automatic language processing depends on imbalanced hemispheric function in schizophrenia.

## Introduction

Schizophrenia (SZ) is one of the mental illnesses characterized by positive symptoms (such as delusions and hallucinations), negative symptoms (such as impaired motivation and social withdrawal), and various cognitive impairments (Owen et al., 2016). A lifetime morbid risk of SZ is approximately 0.7%. Patients with SZ need long-term treatment and care for residual symptoms and cognitive impairments. The clinical stage of SZ is divided into the following: prodrome, first episode, and chronic phase (Wojciak et al., 2016). Regarding cognitive function, patients with SZ have impairments in language, executive function, and attention domains. These cognitive impairments affect their daily functioning, including reasoning or problem-solving skills and socializing, and have been extensively examined in patients with SZ. Some studies have reported that impairment of visual processing in SZ is related to social perception (Corrigan et al., 1994; Sergi and Green, 2003) or social functioning (Sergi et al., 2006; Rassovsky et al., 2011). Visual processing is recognized as one of the major impairments in SZ (Butler et al., 2008; Silverstein and Keane, 2011b); various aspects of visual disturbance, such as deficits of motion perception (Chen, 2011) and perceptual organization (Uhlhaas and Silverstein, 2005; Silverstein and Keane, 2011a), have also been reported.

However, there has been limited investigation of color perception in SZ (Kogata and Iidaka, 2018). Shuwairi et al. (2002) reported that patients with SZ showed impaired color perception in standardized color discrimination tests. Specifically, patients with SZ made several mistakes in distinguishing between red, green, and blue colors; these mistakes differed from those made by patients with dopaminergic dysregulation, such as Parkinson’s disease and cocaine withdrawal (Price et al., 1992; Desai et al., 1997). Conversely, by focusing on the color priming effect, a study by Jahshan et al. (2012) reported that patients with SZ showed a different priming effect compared to healthy controls (HCs). They also suggested that the feed-forward connections of visual relay from the retina to the lateral geniculate nucleus in the thalamus and eventually to the primary visual cortex seem to be intact in patients with SZ. Thus, patients with SZ might have an impairment in color perception due to the disruption of pathways involving secondary or higher visual areas, besides those affecting the primary visual areas.

Furthermore, in a landmark study, David investigated unilateral (i.e., within the left or right visual field) or bilateral (i.e., both left and right visual fields) color recognition in patients with SZ using a tachistoscope (David, 1987). In the color naming task, the author found that patients showed impairment in naming colors upon instant presentation in the left visual field (LVF) condition but not in the right visual field (RVF); this finding suggested that the laterality effect resulted from dysfunction of the interhemispheric connectivity associated with abnormalities in the corpus callosum. In addition, Phillips et al. (1996) investigated the color-Stroop effect using the color-word paring paradigm and revealed that patients with SZ showed stronger color-Stroop facilitation when the stimuli were presented in the LVF than when they were presented in the RVF. Collectively, these findings indicate a lateralized color cognition with respect to cerebral dominance in patients with SZ. However, it remains unclear how the semantic properties of color, such as color name, could affect the performance of color perception.

Regarding the hemispheric laterality, prior studies investigated various lateralities in patients with psychiatric or developmental disorder. Indeed, Berretz et al. (2020) demonstrated atypical hemispheric asymmetries in these patients compared to HCs. Moreover, Berretz et al. (2020) pointed out that these asymmetries were affected by genetic and non-genetic factors. Sommer et al. (2001a) also reported different lateralities between patients with SZ and HCs; the prevalence of handedness, dichotic listening, and anatomical asymmetry. In particular, reduced hemispheric language laterality was identified as a key feature in patients with SZ. Furthermore, Oertel et al. (2010) investigated the laterality in patient with schizophrenia by auditory task and showed that symptom severity was related to a reduced laterality. Thus, atypical hemispheric laterality is an important parameter in patients with SZ.

In the present study, considering the effect of laterality on color recognition, we investigated the performance of patients with SZ and HCs in the color search task using a task developed by Gilbert et al. (2006). This search task manipulated two factors: the target position where the target color square emerges in either the LVF or RVF, and the color category, wherein the target and distractors may have the same or different color names. Since the primary visual cortex in both the hemispheres receives visual information from the contralateral visual field, the left hemisphere should have an advantage in accessing the information appearing in the RVF. In Gilbert et al. (2006)’s study, HCs responded faster in detecting the target in the RVF (i.e., left hemisphere) than in the LVF (i.e., right hemisphere) when the target and distractors had different color names. This laterality effect indicates a significant interaction between hemispheric laterality and lexical processing, which appears to occur in the language-dominant (i.e., left) hemisphere in healthy subjects. Therefore, using this task, we aimed to investigate the effect of laterality and also address the question of how the color lexicon of the target and distractors affect the performance of patients with SZ in the color search task.

In this experiment, we tested two hypotheses. First, patients with SZ would show a slower response when the target was shown in the RVF than when it was shown in the LVF, as reported previously (Posner et al., 1988). Second, the lexical difference in trial condition would affect the search performance depending on the target position. Several studies, using electroencephalogram (Spironelli et al., 2008) and functional magnetic resonance imaging (Sommer et al., 2001b), have demonstrated that language processing was less lateralized in patients with SZ than in HCs, and that the search performance for lexical difference could differ between patients with SZ and HCs. When the target and distractors differ with respect to their color names, patients with SZ would show an advantage (i.e., faster reaction time) when the target is presented in the LVF.

## Materials and Methods

### Participants

This study included patients with SZ (*n* = 16) and HCs (*n* = 16). The patients were hospitalized in a private hospital and met the Diagnostic and Statistical Manual of Mental Disorders, 5th Edition (DSM-5), criteria for SZ. The inclusion criteria for patients were as follows: diagnosis of SZ according to the DSM-5 criteria, diagnosis made more than 3 years ago (i.e., chronic phase) (Fava and Kellner, 1993), no history of brain injury or neurological disorder, and no self-reported color-blindness. Participants with other mental illnesses were excluded. The HCs comprised the staff of a private hospital, who had no mental health problems. The HCs had no brain injury or neurological disorder and no self-reported color-blindness. All participants were native Japanese speakers.

The handedness of all the participants was assessed using the Edinburgh handedness inventory (Oldfield, 1971). In the SZ group, symptoms were measured using the positive and negative syndrome scale (PANSS) (Kay et al., 1987). Social functioning was measured using the social functioning scale (SFS) (Birchwood et al., 1990). The chlorpromazine equivalent dose of prescription drugs was also calculated (Inada and Inagaki, 2015).

The demographic data of both the groups are shown in Table 1. One HC was excluded from the analysis (see results for details). All participants in both the groups were right-handed. There was no significant difference in the mean age of participants between the two groups [*t*(29) = 1.47, *p* = 0.15]. However, there was a significant difference in education years between the groups [*t*(29) = 4.71, *p* < 0.01].

**TABLE 1:** Mean and standard deviation of participants’ demographic characteristics.

	SZ (*n* = 16)	HC (*n* = 15)
Sex (M/F)	10/6	10/5
Age in years	50.6 (5.6)	46.8 (8.4)
Education in years*	13.0 (1.8)	16.1 (1.8)
Chlorpromazine equivalent dose	1,064 (765)	N/A
PANSS		
Positive symptoms	30.4 (3.1)	N/A
Negative symptoms	27.2 (4.1)	N/A
General psychopathology symptoms	61.4 (6.0)	N/A
SFS	50.0 (16.0)	N/A

SZ, schizophrenia; HC, healthy control; PANSS, Positive and negative syndrome scale; SFS, Social functioning scale.*p < 0.05, N/A, not available. SD in parentheses.

This study was approved by the ethics committee of Nagoya University School of Medicine (17–604) and a private hospital. Written informed consent was obtained from all participants.

### Experiment

#### Stimuli

**FIGURE 1:**
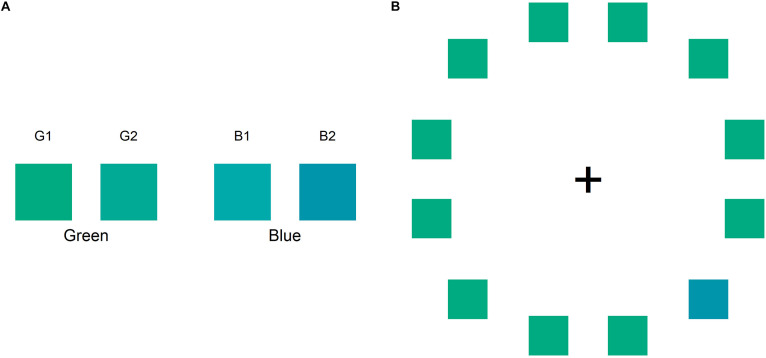
**(A)** Color stimuli used in the color naming and color search tasks: two greens (G1 and G2) and two blues (B1, B2). **(B)** The sample in the color search task comprises a circular ring containing an odd target in the right visual field and 11 distractors.

In this study, we used two visual tasks: the color naming task and color search task. Each of the two tasks took about 5 min to complete. In both the tasks, we used two greenish colors (G1 and G2) and two bluish colors (B1 and B2) as the stimuli (Figure 1A). The experiment was conducted in a dimly lit, quiet room. Participants were seated 50 cm from the screen, with their head fixed on a chin rest. All stimuli were presented on the display (EIZO CG277) and controlled by Presentation version 20.0 (Neurobehavioral Systems, Inc.). The RGB value of the four colors and the background (gray screen) used in the two tasks were as follows: G1 = 0, 171, 129; G2 = 0, 170, 149; B1 = 0, 170, 170; B2 = 0, 149, 170; and background = 170, 170, 170, respectively. Before the experiment, the monitor was calibrated using a built-in color calibration sensor and Color Navigator 6 (EIZO Corporation). In terms of CIE Lab color space, the measured value of the four colors were as follows: G1 = 62.48, –47.68, 11.72; G2 = 62.64, –42.01, –0.71; B1 = 63.24, –35.83, –10.30; and B2 = 56.70, –25.75, –20.35.

#### Color Naming Task

The color naming task was conducted to ensure that the participants in both the groups had similar lexical color-boundaries for the color stimuli used in the color search task. The colors were presented as square objects and shown at a 10° visual angle at the center of the gray screen. Participants were required to classify whether the color was “green” or “blue” by pressing the “G” or “B” key, respectively, on a keyboard. This task included 40 randomized trials (10 trials per color) with no time limit. In the color naming task, the agreement rate was calculated between the color name judged by each participant and the pre-defined setting. The pre-defined setting was as follows: G1 and G2 were labeled as green and B1 and B2 were labeled as blue. Only the participants with more than 50% agreement rate for each color were included in the analysis of the color search task, as described by Gilbert et al. (2006).

#### Color Search Task

The color search task was conducted immediately after the color naming task. This task included six color pairs: B1G1, B1G2, B2G1, B2G2, B1B2, and G1G2. One color was assigned as the odd target color and the others were assigned as the distractors. In this task, participants were required to discriminate between the two colors. The participant discriminated between the odd target and 11 distractors as fast as possible. As shown in Figure 1B, each color search stimulus comprised of 12 colored squares arranged in a circular ring and a central fixation marker (a black cross shown as “ + ”) on a gray screen. The colored squares and the fixation marker were presented at a visual angle of 3°; the circular ring was presented at a visual angle of 16°. In each search stimulus, the target color was either in the LVF or RVF of the central fixation marker. Participants had to judge whether the location of the odd target color was in the LVF or RVF by pressing “Q” or “P” on the keyboard using the left or right index finger, respectively. Each color search trial began with a fixation stimulus comprising only a fixation marker on a gray screen presented for 1,000 ms. In this task, the participants were required to focus on the fixation marker. The color search trial began after the fixation stimulus. When the participants finished performing a search trial, the fixation stimulus was shown again for 250 ms until the next search trial began. The sequence of trial blocks was repeated randomly to counterbalance every condition. Therefore, participants completed 144 random trial blocks in which the target positions (12 positions), color combination (6 pairs), and the target or distractor color (2 patterns) were changed.

The color difference (CIE76) between the two colors was calculated by the “colorscience” package in R (Gama, 2016), which provided a quantitative value of the differences between the two colors. The color difference in each pair was as follows: G1B2 = 39.28, G1B1 = 25.02, G2B2 = 26.18, G1G2 = 13.66, B1B2 = 15.66, and G2B1 = 11.42. The color search task included three trial settings according to the difficulty of perceptual distinction: large color difference (G1B2), middle color difference (G1B1 and G2B2), and small color difference (G1G2, B1B2, and G2B1). Focusing on the color name of the stimuli, the small color difference can be divided into the different-category (G2B1; green and blue) and same-category (G1G2; two greens or B1B2; two blues) conditions on the basis of whether or not the color pairs had the same color lexicon. In the present study, we focused on the small color difference to examine the lexical effect. Therefore, we sorted the trials conducted using small color difference into different-category and same-category conditions to compare the reaction time (RT). In the color search task, the RT data in each trial were excluded if the participant showed error responses or RT outliers. The RT outliers were defined as a response of over or under 2 standard deviations from the mean RT and less than 100 ms.

### Statistical Analysis

In this study, we analyzed the trial data for small color difference. First, to compare the effect of target position in each group, we computed the laterality index of the trial settings in each group and performed a Mann–Whitney U test to assess the laterality indexes of patients with SZ and HC. The laterality index was calculated by using the following formula:


L⁢a⁢t⁢e⁢r⁢a⁢l⁢i⁢t⁢y⁢i⁢n⁢d⁢e⁢x=[R⁢T⁢(L⁢V⁢F-R⁢V⁢F)×100/R⁢T⁢(L⁢V⁢F+R⁢V⁢F)](O’Regan and Serrien, 2018).

Second, to compare the interaction between color category and target position, we also computed the categorical perception (CP) index in the LVF/RVF in each group and performed a Wilcoxon signed-rank test for the CP indexes of patients with SZ and HCs. The CP index in LVF/RVF was calculated by the following formula:


C⁢P⁢i⁢n⁢d⁢e⁢x=[R⁢T⁢(s⁢a⁢m⁢e-c⁢a⁢t⁢e⁢g⁢o⁢r⁢y-d⁢i⁢f⁢f⁢e⁢r⁢e⁢n⁢t-c⁢a⁢t⁢e⁢g⁢o⁢r⁢y)×100/R⁢T⁢(s⁢a⁢m⁢e-c⁢a⁢t⁢e⁢g⁢o⁢r⁢y+d⁢i⁢f⁢f⁢e⁢r⁢e⁢n⁢t-c⁢a⁢t⁢e⁢g⁢o⁢r⁢y)].

In addition, for patients with SZ, we computed Pearson’s correlation coefficients to assess for potential correlations between the mean RT and patients’ characteristics, including chlorpromazine equivalent dose, PANSS, and SFS. We also calculated the adjusted *p*-value to control multiple comparisons using a false discovery rate in the analysis of Pearson’s correlation coefficients (Benjamini and Hochberg, 1995).

All statistical analyses were performed using R version 3.4.4. The statistical threshold was set at *p* < 0.05 in all analyses.

## Results

### Color Search Task

The mean accuracy rate of the SZ and HC groups in the color search task was 98% (range, 87–100%) and 98% (range, 94–100%), respectively. Except for the erroneous answers and a deviated RT, we used 93% of the data (range, 85–97%) for the SZ group and 93% of the data (range, 89–96%) for the HC group for further analysis. The RT data for one HC participant were excluded because they were greater than the third quartile plus 1.5 times the interquartile range in the small color difference condition. Therefore, the data for 16 patients with SZ and 15 HCs were analyzed further.

**FIGURE 2:**
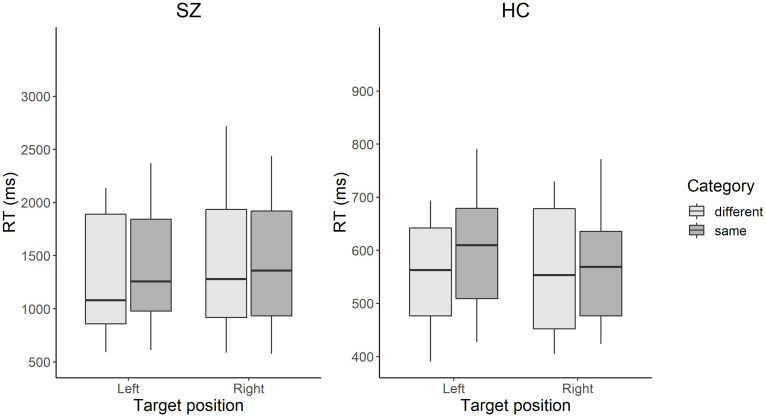
Box plots of RT in each condition in patients with SZ and HCs. The color search task manipulated two factors: (1) the position from which the target colored square emerges: left visual field or the right visual field; and (2) the color-category condition in which the target and distractors have the same color name (e.g., two different blues) or different color names (e.g., a blue and a green). The box represents the range between the lower and upper quartiles. The line in the box represents the median. The whisker represents the range between the minimum and maximum values. Scales in the vertical axes differ between the groups. SZ, patients with schizophrenia; HC, healthy controls.

The grand mean RTs, including those in all the trial settings of large, middle, and small color difference, were 1,235 ms (*SD* = 456) for the SZ group and 531 ms (*SD* = 89) for the HC group. Table 2 shows the mean RT in each condition, grouped only by the small color difference condition. Figure 2 shows the RT results in each condition for the interaction between target position and category.

**TABLE 2:** Mean RT (ms) in each condition for patients with schizophrenia and healthy controls.

	SZ (*n* = 16)			HC (*n* = 15)		
	Mean	*SD*	95% CI	Mean	*SD*	95% CI
LVF						
Different-category	1,276	554	981, 1,572	559	102	502, 615
Same-category	1,414	552	1,120, 1,708	593	115	529, 656
RVF						
Different-category	1,442	663	1,089, 1,795	565	122	498, 632
Same-category	1,436	579	1,128, 1,745	576	118	511, 642

*SZ, schizophrenia; HC, healthy control; LVF, left visual field; RVF, right visual field.*

Regarding the target position, the laterality index for RT was calculated in each group. The positive and negative values of laterality index refer to slower responses when the target emerged in the LVF and RVF, respectively. The mean laterality index was –1.3 (*SD* = 2.6) for the SZ group and 1.4 (*SD* = 3.8) for the HC group. There was a significant difference between the laterality index of the SZ and HC groups [*z* = 2.22, *p* = 0.03, *r* = 0.40], indicating that patients with SZ showed slower responses in the RVF than HCs.

**FIGURE 3:**
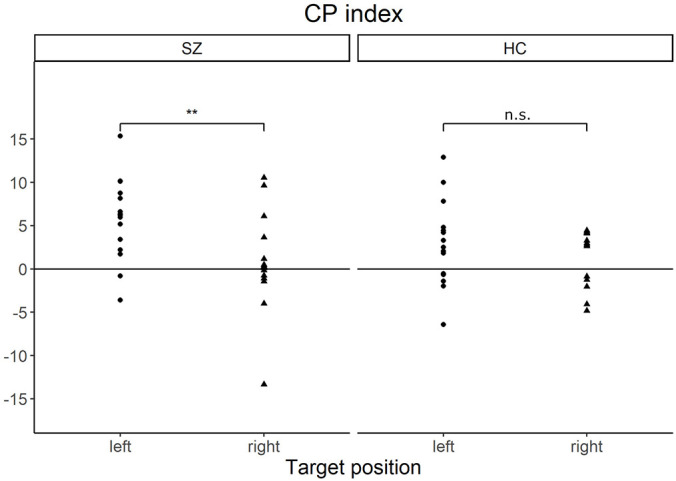
Scatter plots of the CP index in each target position of patients with SZ and HCs. The CP index indicates how the color-category condition affects visual search performance. The positive value of CP index indicates that the RT is faster in the different-category conditions than in the same-category conditions. Patients with SZ showed a significantly greater CP index in the left visual field than in the right visual field. **p < 0.01, n.s., not significant; CP, categorical perception; SZ, patients with schizophrenia; HC, healthy controls.

Furthermore, we focused on the CP indexes for RT in the LVF/RVF in each group to examine the effect of target position (LVF or RVF) and category (different-category or same-category). A positive value of CP index indicates faster response in the different-category condition than in the same-category condition (positive CP effect). Conversely, a negative value of CP index indicates a faster response in the same-category condition than in the different-category condition (negative CP effect). Figure 3 shows the laterality of the CP indexes in both the groups. In patients with SZ, the mean CP indexes were 5.8 (*SD* = 4.6) for the LVF and 0.7 (*SD* = 5.5) for the RVF. In HCs, the mean CP indexes was 2.9 (*SD* = 4.9) for the LVF and 1.1 (*SD* = 3.2) for the RVF. In terms of the CP index, patients with SZ showed a significantly greater CP effect in the LVF than in the RVF [*z* = 3.00, *p* = 0.004, *r* = 0.28], whereas HCs did not show significant laterality [*z* = 1.08, *p* = 0.28].

### Correlation Between Search Performance and Clinical Data

The relationship between visual search performance and clinical data was analyzed by Pearson’s correlation coefficients and the results are shown in Table 3. There were some significant or marginally significant correlations between the grand mean RTs, including those of all trial settings (large, middle, and small color differences), and negative symptoms [*r*(14) = 0.67, adjusted *p* = 0.02], general psychopathology symptoms [*r*(14) = 0.54, adjusted *p* = 0.08] in the PANSS, and SFS [*r*(14) = –0.50, adjusted *p* = 0.08].

**TABLE 3:** The relationship between measures and grand mean RT in patients with schizophrenia.

Measure	*r*	Adjusted *p*
Chlorpromazine equivalent dose	–0.10	0.89
PANSS positive symptoms	0.03	0.92
PANSS negative symptoms	0.67	0.02
PANSS general psychopathology symptoms	0.54	0.08
SFS	–0.50	0.08

*PANSS, Positive and negative syndrome scale; SFS, Social functioning scale.*

## Discussion

In this study, we examined the visual search performance for color perception in patients with chronic SZ and HCs. This study aimed to investigate how the position of the target and the color category of the target and distractors affected visual search performance. Differences in visual search performance were previously identified in normal adults (Gilbert et al., 2006), children (Franklin et al., 2008), and patients with aphasia (Paluy et al., 2011). In subjective reports and semi-structured interviews, patients with SZ reported distorted color perception (Chapman, 1966; Cutting and Dunne, 1986; Vollmer-Larsen et al., 2007). However, there is little evidence of impairment of color discrimination performance in patients with SZ, except in color perception (Shuwairi et al., 2002) and color priming tasks (Jahshan et al., 2012). Therefore, to the best of our knowledge, the present study is the first to elucidate lateralized color search performance and the relationship between target position and color categorical effect in SZ.

The present study revealed two main results: first, patients with SZ showed a significantly slower RT when the target emerged in the RVF than when it emerged in the LVF; and second, regarding the interaction effect of target position and color category, patients with SZ had differential search performance when compared with HCs. In particular, regarding the CP effect, patients with SZ were quicker to detect the target in the LVF than in the RVF when both the target and distractors had different color names. This finding indicates that, in patients with SZ, semantic processing for color name differences influenced visual discrimination performance more greatly in the LVF than in the RVF. However, the same effect was not observed in HCs. These findings suggest that patients with SZ had a different search performance when compared to HCs, which appears to relate to language lateralization in the dominant left hemisphere.

Regarding visual field, several studies have reported asymmetric performance in patients with SZ, such as a slower response in the RVF (Posner et al., 1988; Matsuda et al., 2011) and more omissions in the RVF (Liu et al., 2011), both of which indicate left hemispheric dysfunction. Our results of a significant difference in laterality index show that patients with SZ detected targets more slowly in the RVF than in the LVF and replicated the tendencies for inattention in the RVF reported previously (Posner et al., 1988; Matsuda et al., 2011). The asymmetry of task performances, which suggests left hemispheric dysfunction in SZ, is supported by a visual processing task conducted using either the LVF or RVF (Gur, 1978).

Conversely, the significant visual field difference in CP effect was observed only in the SZ group, indicating a lateralized effect of semantic properties in the color search task in SZ. In patients with SZ, the CP effect, wherein the response of color name difference between the target and distractors was faster in the search task, was observed more strongly in the LVF than in the RVF. In HCs, there was no laterality effect in the current study, which is similar to a previous report by Witzel and Gegenfurtner (2011). The left-lateralized CP effect for color search was also observed in patients with left hemisphere injury (i.e., patients with aphasia) (Paluy et al., 2011). In addition, this laterality effect could vary according to the level of color name acquisition during developmental stages (Franklin et al., 2008). Children who acquired color name showed a right-sided CP effect, whereas children who did not acquire color name showed a left-sided CP effect; the shift in laterality occurred during the process of typical development. Since these findings are relevant to language disintegration in patients with brain-damage and normally developing children, it is suggested that the laterality of the CP effect could be based on plasticity of the brain circuits.

Furthermore, some studies investigated brain activation in HCs who performed a color search task (Liu et al., 2009; Siok et al., 2009). In particular, Siok et al. (2009) suggested that some language regions might play an important role in manipulating top-down control in the color search task. Although the precise mechanism underlying this search performance is unknown, color stimuli could easily activate regions related to color name in the process of detecting the target color. Richter and Zwaan (2009) revealed that the processing of color perception and color naming have overlapping representational resources. This study showed that presentation of a matching color word facilitates quick color discrimination, suggesting that color perception and naming involve similar underlying processes.

A classical study on SZ reported notable descriptions of color categorization (Bolles and Goldstein, 1938). In this study, some patients with SZ refused to classify various shades of green into green color as a whole, indicative of unstable or variant color categorization schema in the brain. In SZ, these findings have been characterized as thought disorder or language disturbances linked to semantic performance (Doughty and Done, 2009). Regarding left hemisphere dysregulation of language, Crow postulated that patients with SZ show different hemispheric specialization for language (Crow, 1997). Crow also suggested that some deterioration, such as semantic deficits, were critical language failures related to hemispheric specialization. Subsequently, Li et al. (2009) suggested that, compared with HCs, left hemisphere dominance of language is generally disturbed in patients with SZ, and that heterogeneity in hemispheric lateralization could underlie psychiatric symptoms. Previous studies regarding language lateralization investigated hemispheric activation with a verbal task, such as a listening task (Koeda et al., 2006; Razafimandimby et al., 2007; Dollfus et al., 2008) or word fluency task (Boksman et al., 2005). Although some studies investigated brain activation using a visual task, such as semantic judgement (Kubicki et al., 2003; Li et al., 2007), only few studies have used semantic tasks with a manipulated visual field. Therefore, further research is needed to investigate the relationship between the lateralized visual task regarding semantic features and brain structure or activation in SZ, which may involve large-scale brain networks related to semantic processing.

Some significant or marginally significant correlations concerning the relationship between search performance and patients’ characteristics were also observed in this study. In particular, the slower RT was related to lower social functioning and more frequent negative and psychopathological symptoms. Furthermore, consistent with the findings of earlier studies, the present study results showed that visual processing was related to functional outcome (Sergi et al., 2006; Rassovsky et al., 2011). Conversely, there was no significant correlation between search performance and chlorpromazine equivalent dose, suggesting that the search performance did not depend on medication use.

Despite the strengths, this study has some limitations. The first limitation is the small sample size. Therefore, in the future, to validate the results of the present study, it is necessary to acquire data with a sufficiently large number of subjects. The second limitation is that we did not confirm the participants’ color blindness by conducting a precise medical evaluation. The finding that all participants performed the color naming task and color search task with high accuracy may, however, preclude such a possibility. Furthermore, this study did not measure eye movement, which might affect search performance.

## Conclusion

In conclusion, this study presents two main findings. First, patients with SZ responded more quickly to targets in the LVF than those in the RVF. This laterality effect suggests an advantage for the right hemisphere or disadvantage for the left hemisphere in the visual task in patients with SZ. Second, for patients with SZ, the categorical condition influenced color search performance depending on target position. In the different-category condition, patients with SZ responded more quickly when the target was in the LVF than when it was in the RVF. A similar tendency was not observed in HCs. This lateralized performance could imply failure of left hemisphere dominance in language processing in SZ. A search paradigm combining both the category and target position factors may clarify the mechanism of language in automatic processing and lead to further examination of imbalanced hemispheric function in SZ.

## Data Availability Statement

The original contributions presented in the study are included in the article/supplementary material, further inquiries can be directed to the corresponding author/s.

## Ethics Statement

This research was approved by the Ethics Committee of Nagoya University School of Medicine (17–604). The patients/participants provided their written informed consent to participate in this study.

## Author Contributions

TK collected, analyzed and interpreted the data, and wrote the manuscript. TI interpreted the data and also wrote the manuscript. Both authors were involved in drafting the manuscript, reading and approving the final manuscript.

## Conflict of Interest

The authors declare that the research was conducted in the absence of any commercial or financial relationships that could be construed as a potential conflict of interest.

## Publisher’s Note

All claims expressed in this article are solely those of the authors and do not necessarily represent those of their affiliated organizations, or those of the publisher, the editors and the reviewers. Any product that may be evaluated in this article, or claim that may be made by its manufacturer, is not guaranteed or endorsed by the publisher.
